# Hypophosphatemic Osteomalacia Induced by Low-Dose Adefovir-Related Fanconi Syndrome

**DOI:** 10.14740/jmc5281

**Published:** 2026-02-02

**Authors:** Dohee Kim

**Affiliations:** Department of Internal Medicine, Division of Endocrinology, Dankook University College of Medicine, Cheonan 330–714, Korea. Email: dh9070@hanmail.net

**Keywords:** Adefovir dipivoxil, Fanconi syndrome, Hypophosphatemic osteomalacia

## Abstract

Adefovir dipivoxil (ADV), an antiviral agent for chronic hepatitis B virus infection, has been associated with nephrotoxicity, particularly Fanconi syndrome, even at low doses (10 mg/day). Fanconi syndrome is a generalized proximal tubular dysfunction leading to phosphate wasting and hypophosphatemic osteomalacia. Here, the author reports a case of a 58-year-old man with a 2-year history of bone pain involving the left foot, upper back, and left scapula, later extending to the right foot and chest wall, without antecedent trauma. Magnetic resonance imaging revealed insufficiency fractures in both calcanei and healing fractures of the spinous processes from the sixth cervical to the second thoracic vertebrae with callus formation. Whole-body bone scintigraphy demonstrated increased uptake in multiple ribs, the lower sacral region, and the aforementioned areas. Imaging findings, together with elevated serum alkaline phosphatase with increased bone fraction, prompted an extensive malignancy workup, which was negative. The patient had been taking ADV 10 mg daily for 10 years for chronic hepatitis B. Laboratory evaluation showed mild renal dysfunction, hypophosphatemia, hypouricemia, elevated alkaline phosphatase, proteinuria, phosphaturia, and glucosuria without hyperglycemia. He was diagnosed with hypophosphatemic osteomalacia secondary to ADV-induced Fanconi syndrome. After switching from ADV to entecavir and initiating supplementation with calcitriol and phosphate, the patient’s symptoms and laboratory abnormalities improved significantly. Careful monitoring of serum phosphate, alkaline phosphatase, and renal function is essential for early recognition and timely intervention in these potentially reversible adverse drug reactions.

## Introduction

Adefovir dipivoxil (ADV) has been widely used in the management of chronic hepatitis B virus (HBV) infection [1]. Although renal toxicity associated with ADV is dose-dependent and the recommended low dose of 10 mg/day is generally considered safe, recent case reports and series have indicated that even at this low dose, prolonged use may lead to proximal renal tubular dysfunction, known as Fanconi syndrome, particularly in East Asian populations [1–3]. Fanconi syndrome is a proximal renal tubular disorder characterized by the loss of phosphate, bicarbonate, uric acid, glucose, and amino acids, which can subsequently result in hypophosphatemic osteomalacia. The most frequent clinical symptoms of osteomalacia include nonspecific bone pain, muscle weakness, and an increased risk of fractures. Because of its insidious onset, varied clinical manifestations, nonspecific radiologic findings, and non-characteristic routine serum biochemical changes, the disease is often mistaken for other musculoskeletal conditions, and diagnosis may be delayed or misinterpreted [3–5].

The author reports a rare case of hypophosphatemic osteomalacia with Fanconi syndrome caused by long-term low-dose ADV, which was initially investigated for malignancy because of multiple hot uptakes on whole-body bone scintigraphy and elevated serum alkaline phosphatase (ALP) bone fraction levels.

## Case Report

### Investigations

**Figure 1:**
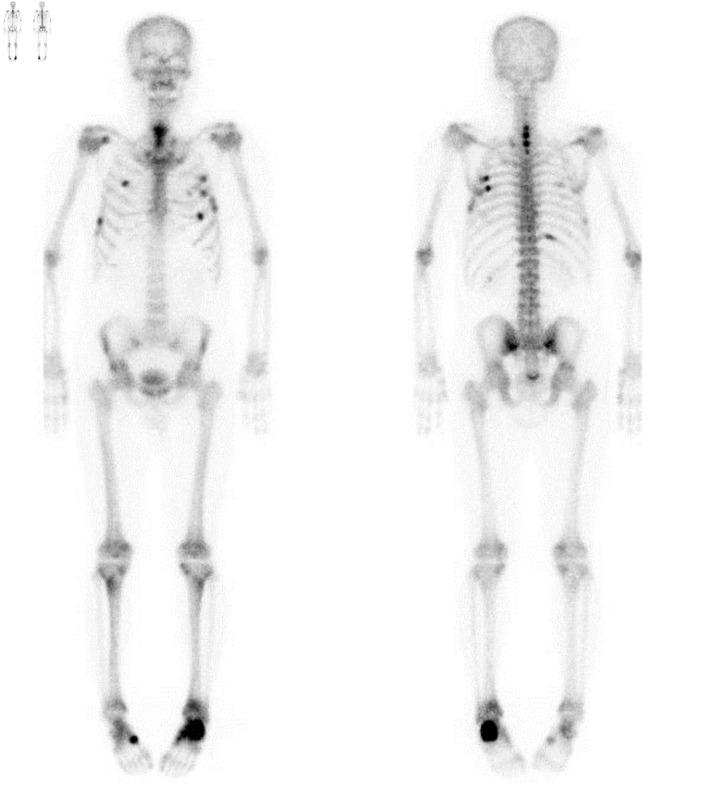
Whole-body bone scintigraphy showing multiple areas of increased radiotracer uptake involving the anterior arcs of the right third and seventh ribs, the posterior arcs of the right sixth and 11th ribs, the anterolateral and posterior arcs of the left third through sixth ribs, the C5–T1 spine, the left calcaneus, the right midfoot, and the lower sacral region.

A 58-year-old man was referred to the Endocrinology Department in September 2022 for evaluation and treatment of very low bone mineral density (BMD) (T-score of −4.5 in the lumbar spine) and a 2-year history of bone pain involving both feet, the upper back, the left scapula, and the chest wall, without antecedent trauma. He had first been referred to the Orthopedic Department of our clinic in January 2021 because of left heel pain lasting 3 months, with magnetic resonance imaging (MRI) from another local clinic showing a fracture line along the physis of the calcaneal posterior tuberosity. The patient, a physician by occupation, had been walking about 10 km daily on his commute as exercise, until he stopped in October 2020 due to heel pain while walking. He also reported pain in the upper back and along the medial border of the left scapula for 1–2 years, which had been diagnosed as myofascial pain syndrome at another local clinic. On physical examination, there was mild tenderness over the posterior tuberosity of the left calcaneus with local warmth in the left heel, as well as tenderness along the medial border of the left scapula, with pain aggravated by abduction of the left arm. Plain radiographs of both feet, the scapula, and the spine demonstrated an incomplete or stress fracture in the posterior body of the left calcaneus, multiple rib fractures with immature callus formation in the left upper ribs, and mild degenerative changes with osteophytes, respectively. Whole-body bone scintigraphy using technetium-99m methylene diphosphonate (^99m^Tc-MDP) revealed multiple areas of increased uptake along the anterior arcs of the right third and seventh ribs, posterior arcs of the right sixth and 11th ribs, anterolateral and posterior arcs of the left third to sixth ribs, the C5–T1 spine, the left calcaneal area, the right midfoot, and the lower sacral region, suggesting multiple fractures or possible bone metastases (Fig. 1). Cervical spine MRI demonstrated fracture healing of the C6–T2 spinous processes with callus formation. The patient’s serum ALP level was elevated, with an increased bone fraction. Extensive malignancy workup—including blood tests (tumor markers and protein electrophoresis), urine protein electrophoresis, positron emission tomography–computed tomography, chest and abdominal computed tomography, and esophagogastroduodenoscopy—was negative. Thereafter, his left heel pain improved, but he developed pain in the right heel. Bilateral foot MRI performed in August 2022 at a local clinic showed healing of the left calcaneus and a stress fracture in the right calcaneus, findings suggestive of metabolic bone disease. Laboratory results from the same clinic 1 month before presentation (August 2022) were as follows: serum ALP 297 U/L (reference 40–129; 81.7% bone fraction), ionized calcium 1.0 mmol/L (1.10–1.34), intact parathyroid hormone (PTH) 34.1 pg/mL (15.0–65.0), 25-hydroxyvitamin D (25(OH)D) 20.7 ng/mL (vitamin D insufficiency 10.0–30.0), procollagen type 1 N-terminal propeptide (P1NP) 86 ng/mL (22.9–85.3), C-terminal telopeptide of type 1 collagen (CTx) 1.190 ng/mL (0.161–0.737), hemoglobin A1c 6.0%, and lumbar spine BMD T-score −4.5 (L1–4). Thyroid hormone, cortisol, testosterone, complete blood count, and liver and renal function tests were within normal limits, except for low uric acid. Urinalysis revealed protein 2+ and glucose 4+.

At his initial presentation to the Endocrinology Department, the patient had a medical history of type 2 diabetes mellitus, hypertension, and dyslipidemia since 2018, for which he had been taking empagliflozin 10 mg, telmisartan 80 mg, atorvastatin 10 mg, and aspirin 100 mg daily. He was also been diagnosed with HBV infection in May 2000 and chronic hepatitis B, and started on lamivudine therapy in August 2000. On the presentation, he had been on adefovir 10 mg daily for 10 years in addition to lamivudine 100 mg daily. He had undergone surgery for patent ductus arteriosus in 1975. On examination, his height was 171.0 cm and weight 72.0 kg. Blood pressure was 126/84 mm Hg, and heart rate 76 beats per minute.

### Diagnosis

Laboratory data on presentation are shown in Table 1. He demonstrated hypophosphatemia, hypouricemia, slightly low hypocalcemia, increased levels of serum ALP and creatinine, mild hyperglycemia and hypermagnesemia, vitamin D insufficiency and reduced estimated glomerular filtration rate (eGFR). Serum intact PTH, 1,25-dihydroxyvitamin D (1,25(OH)_2_D), aspartate transaminase, alanine transaminase, sodium, and potassium were within normal limits. Urinalysis showed proteinuria and glycosuria with fasting blood glucose of 138 mg/dL. The patient’s 24-h urine results revealed proteinuria, hypercalciuria, and hyperphosphaturia with decreased tubular reabsorption of phosphorus (TRP) 34.0% (85–95%) and tubular maximum reabsorption of phosphate/GFR 1.79 mg/dL (2.6–3.8), indicating generalized proximal tubular reabsorptive dysfunction, including renal phosphate wasting. BMD (dual-energy X-ray absorptiometry, Hologic) showed a T-score and Z-score of −2.4 and −1.3, respectively, in the lumbar spine (L1–4); −2.3 and −1.4 in the femoral neck; and −2.2 and −1.8 in the total hip, indicating osteopenia (Table 1).

**Table 1:** Laboratory Values at the Initial Presentation to the Endocrinology Department and During Follow-Up

Laboratory test (reference range)	Initial	3 mo	6 mo	1 yr	2 yr	3 yr
Serum						
Calcium (8.6–10.2 mg/dL)	9.1	9.2	9.8	9.2	9.3	9.3
Albumin (3.5–5.2 g/dL)	5.3	4.5	5.1	4.8	4.9	4.6
Corrected calcium (8.6–10.2 mg/dL)	8.1^a^	8.8	8.9	8.6	8.6	8.8
Phosphorus (2.5–4.5 mg/dL)	1.8^a^	2.8	3.1	2.86	3.2	3.1
Alkaline phosphatase (40–129 U/L)	308^a^	419^a^	283^a^	193^a^	109	105
Creatinine (0.67–1.17 mg/dL)	1.31^a^	1.23^a^	1.33^a^	1.26^a^	1.22^a^	1.14
Estimated glomerular filtration rate (mL/min/1.73 m^2^)	60	64	58	62	67	73
Fasting glucose (70–99 mg/dL)	138^a^		148^a^	125^a^	117^a^	136^a^
Hemoglobin A1c (%)			6.1^a^	6.2^a^	6.4^a^	6.9^a^
Uric acid (3.4–7.0 mg/dL)	1.8^a^		2.7^a^	2.8^a^	3.1^a^	2.8^a^
Intact parathyroid hormone (8–76 pg/mL)	14.4		12.8	12.8	12.1	13
25-hydroxyvitamin D (ng/mL)	21.7^a^		31.4	34.5	31.7	34.8
1,25-dihydroxyvitamin D (19.6–54.3 pg/mL)	47.35		81.49^a^	82.99^a^	76.68^a^	78.97^a^
Magnesium (1.6–2.4 mg/dL)	2.44^a^			2.41^a^	2.28	2.29
Aspartate aminotransferase (0–40 IU/L)	15		18	17	25	19
Alanine aminotransferase (0–40 IU/L)	14		20	16	24	19
Sodium (136–145 mmol/L)	138	139	139	138	138	136
Potassium (3.5–5.1 mmol/L)	4.4	4.5	4.5	4.3	4.3	4.1
Chloride (98–107 mmol/L)	105	105	104	104	104	103
Urine analysis						
Protein	2+^a^			1+^a^	1+^a^	1+^a^
Glucose	3+^a^			3+^a^	3+^a^	3+^a^
24-h urine laboratory						
Protein (0–150 mg/day)	58					
Calcium (100–300 mg/day)	328					
Phosphorus (0.4–1.3 g/day)	0.95					
Creatinine (1.04–2.35 g/day)	1.05					
Tubular reabsorption of phosphorus (85–95%)	34^a^					
Spot urine						
Microalbumin (mg/dL)				11.2	12.3	10.4
Calcium (mg/dL)				35.7	30.6	21.1
Phosphorus (mg/dL)				69.28	78.6	58.6
Creatinine (39–259 mg/dL)				119.7	117.2	87.6
Microalbumin/creatinine ratio (mg/g)				94^a^	104^a^	119^a^
Calcium/creatinine clearance ratio				0.04	0.037	0.029
Tubular reabsorption of phosphorus (85–95%)				74.4^a^	74^a^	75.4^a^
BMD by DXA measurement, T-score						
Lumbar spine (L1–4)	–2.4^a^			–1.1^a^		–0.9
Femoral neck	–2.3^a^			–1.4^a^		–1
Total hip	–2.2^a^			–0.7		–0.5

^a^Abnormal values. 3 mo: 3 months; 6 mo: 6 months; 1 yr: 1 year; 2 yr: 2 years; 3 yr: 3 years; BMD: bone mineral density; DXA: dual-energy X-ray absorptiometry.

Given these results, a diagnosis of hypophosphatemic osteomalacia secondary to adefovir-induced Fanconi syndrome was made.

### Treatment

In consultation with the patient’s hepatologist, adefovir was switched to entecavir. The patient was started on oral phosphate 1 g daily and calcitriol 0.25 µg daily. After 7 weeks of supplementation and the change from adefovir to entecavir, his serum phosphate level rose to 2.8 mg/dL, and the bone pain was markedly improved. Oral phosphate supplementation was then reduced to 500 mg daily, and calcitriol was replaced with cholecalciferol 1,000 IU and calcium 500 mg daily. After an additional month, the bone pain had completely resolved and oral phosphate was discontinued, with normalization of serum phosphate and calcium levels. At the 1-year follow-up, laboratory results showed normal serum calcium, phosphate, 25(OH)D, and intact PTH, although elevated serum ALP, hypouricemia, decreased TRP, proteinuria, low BMD remained abnormal but were much improved (Table 1).

### Follow-up and outcomes

During 3 years of follow-up, the patient reported no recurrence of bone pain; maintained normal serum calcium, phosphate, and ALP levels; and demonstrated normal BMD while continuing cholecalciferol 1,000 IU and calcium 500 mg daily (Table 1).

## Discussion

ADV is a nucleotide analogue of adenosine monophosphate that is metabolized *in vivo* to the active form, adefovir diphosphate, which acts as an anti-HBV drug by inhibiting HBV DNA polymerase and reverse transcriptase [6]. ADV-induced nephrotoxicity is dose-dependent: while significant toxicity occurs at high doses (> 30 mg/day), low-dose therapy was previously considered safe. However, subsequent studies have shown a high prevalence of renal toxicity with prolonged low-dose administration, with the 5-year incidence of nephrotoxicity at 10 mg/day ranging from 3% to 8% [4, 6, 7]. ADV-induced nephrotoxicity is characterized by generalized proximal tubular dysfunction—manifesting as Fanconi syndrome—and impaired reabsorption of essential solutes, particularly phosphate, leading to phosphaturia and hypophosphatemia. Chronic phosphate depletion hinders bone mineralization and results in osteomalacia. Additional urinary losses of bicarbonate and other solutes such as amino acids, glucose, uric acid, calcium, or magnesium further aggravate the clinical presentation [3].

Although the mechanism of ADV-induced renal injury is not fully understood, transporter proteins in the renal tubule may contribute to excessive drug accumulation in tubular epithelial cells. Adefovir enters proximal tubular epithelial cells from the bloodstream via the human organic anion transporter 1 (hOAT1), located on the basolateral membrane, and is then secreted into the tubular lumen and excreted in the urine through multidrug-resistant proteins on the apical membrane of renal tubular cells [1, 6, 7]. Long-term or high-dose ADV administration can lead to intracellular accumulation of the drug, which exerts mitochondrial toxicity by inhibiting mitochondrial DNA polymerase in proximal tubular cells and suppressing mitochondrial DNA synthesis. Ultrastructural studies of the kidney have demonstrated enlarged and deformed mitochondria, reduced mitochondrial DNA content, cytochrome oxidase deficiency, and impaired oxidative and respiratory function. This mitochondrial toxicity and inhibition of mitochondrial function can ultimately result in proximal tubular damage and apoptosis of renal tubular epithelial cells [1, 6].

An increasing number of case reports and series have described low-dose ADV–induced Fanconi syndrome or hypophosphatemic osteomalacia, most frequently in East Asian populations. Renal impairment during long-term ADV treatment for HBV has been reported in 20.2% of Chinese patients, 10.5% of Korean patients [6], and 9.6% of Japanese patients [2]. Possible explanations include the higher prevalence of HBV infection—and consequently, more frequent use of adefovir—in East Asia; a relatively lower body mass index in this population, identified as a risk factor in a Japanese study; and potential genetic predispositions, such as polymorphisms in the *hOAT1* gene and multidrug-resistant protein 2 [6, 8].

The main clinical presentations of Fanconi syndrome or hypophosphatemic osteomalacia caused by long-term adefovir therapy include fatigue, progressive systemic pain in multiple bones and joints, difficulty walking, and pathological fractures [4, 6, 7]. Most patients presented with gradually worsening bone pain at weight-bearing sites, primarily beginning in the heel and then spreading to the lower limbs, lower back, and ribs [6, 7]. Laboratory findings revealed hypophosphatemia, elevated serum ALP, hypouricemia, elevated serum creatinine, reduced GFR, proteinuria, nondiabetic glycosuria, metabolic acidosis, elevated bone turnover markers, and osteoporosis [4, 6, 7]. Most patients were between 41 and 70 years of age (84.5%; range 22–72 years), with a male-to-female ratio of 2.9:1 [6]. The median duration of ADV treatment before symptom onset was 52 to 60 months (range 12–132 months) [6, 7]. The average total duration of medication was approximately 18.2 months longer than the time to symptom onset, indicating delays in correct diagnosis after symptoms had appeared [6]. There were no significant differences—other than sex—between the Fanconi syndrome group and the group with Fanconi syndrome plus hypophosphatemic osteomalacia [6]. The predictive factors for adefovir-induced kidney damage were an age of ≥ 40 years, residence in rural areas, prior renal toxicity, eGFR of < 90 mL/min/1.73 m^2^, hypertension, diabetes, cirrhosis, and duration of adefovir treatment exceeding 24 months [4].

In this case, it took approximately 23 months from the onset of bone pain to the diagnosis of osteomalacia, despite typical clinical features such as bone pain, elevated serum ALP levels, insufficiency fractures, and distinctive patterns on whole-body scans. This is consistent with other case studies, which reported diagnostic delays of 6–33 months [3, 6, 8]. Prior to diagnosis, the patient had also undergone multiple examinations, including extensive workups for malignancy. A retrospective study reported that 14 of 28 patients (50%) continued ADV treatment after the onset of joint symptoms, with a mean delay of 21.3 months. These patients visited multiple medical facilities, underwent repeated testing, and received ineffective treatment before the correct diagnosis was made [4, 9]. In a study of 76 patients who developed ADV-induced hypophosphatemic osteomalacia, several patients were initially misdiagnosed with osteoporosis (13 patients), spondyloarthropathy (three patients), or multiple myeloma (one patient), and bone metastases (four patients) due to multiple increased uptakes in whole-body bone scans [7]. Chen et al [10] also reported misdiagnosis of ADV-induced Fanconi syndrome in 27 of 120 patients, most commonly as osteoporosis (18.0%), ankylosing spondylitis (16.0%), vertebral disc herniation (12.0%), osteoarthropathy (10.0%), and bone tumors (10.0%) [9, 10]. Bone pain from osteomalacia is often confused with musculoskeletal complications of osteoporosis, such as insufficiency fractures, or vertebral compression fractures. Moreover, osteoporosis itself is common, affecting up to 50% of patients with chronic liver disease [3]. Reported prevalence ranges from 20% to 53%, with fracture rates up to 11%, particularly higher among those treated with tenofovir disoproxil fumarate (TDF), another antiviral agent for chronic HBV infection. TDF-induced bone loss of 4–5% occurs at the spine and hip during the first 12 months of treatment and stabilizes thereafter [11]. Long-term administration of ADV or TDF in patients with chronic hepatitis B can lead to decreased renal function and reduced BMD. According to the Korean clinical guideline, if patients have preexisting risk factors for renal dysfunction and/or metabolic bone disease, or if worsening kidney function or bone disease is detected during treatment, therapy should be switched to tenofovir alafenamide fumarate, entecavir, or besifovir, with appropriate dose adjustment based on creatinine clearance [12].

Osteomalacia is challenging to diagnose for several reasons. Diagnosis is often delayed because of its insidious onset and nonspecific symptoms such as bone pain and fatigue. Because of variable clinical manifestations, nonspecific radiological findings, and non-characteristic routine serum biochemical changes, osteomalacia is frequently mistaken for musculoskeletal diseases such as osteoarthrosis, osteoporosis, or bone tumors in its early stages. In addition, serum phosphorus levels are not routinely included in standard blood chemistry panels. Hypophosphatemic osteomalacia is also less common than vitamin D–deficiency–related osteomalacia and is therefore less familiar to clinicians [3–5, 8, 13]. A high index of suspicion is essential for early diagnosis. In a retrospective case series of adult-onset hypophosphatemic osteomalacia as a cause of widespread musculoskeletal pain at a single tertiary center [5], six of eight patients were ultimately diagnosed with ADV-induced Fanconi syndrome. Clinical features such as mechanical pain characteristics, insufficiency fractures, proximal muscle weakness, hypophosphatemia, and distinctive bone scintigraphy patterns—including the “adult rachitic rosary,” pseudoreactivation of the growth plate, and the “tie sign” of the sternum—can serve as early diagnostic indicators [5].

Laboratory findings typically associated with hypophosphatemic osteomalacia include markedly decreased serum phosphate, normal or slightly low serum calcium, increased urinary phosphate, reduced renal phosphate threshold, elevated ALP, and normal or mildly elevated PTH [14]. In this case, 24-h urinalysis revealed both hypercalciuria and hyperphosphaturia, along with mild hypocalcemia. Hypercalciuria is common in adefovir-induced nephrotoxicity and may result from proximal tubular damage, although calciuria may be normal depending on the severity of the lesion [3]. At the time of diagnosis, the patient had slightly low serum calcium with inappropriately normal PTH, along with vitamin D insufficiency and inappropriately normal active vitamin D, suggesting possible primary hypoparathyroidism. It is hypothesized that vitamin D insufficiency, combined with skeletal resistance to PTH and underlying osteomalacia, contributed to an inadequate calcemic response [15]. During follow-up, 1,25(OH)_2_D levels remained consistently elevated, while calcium, phosphate, 25(OH)D, and PTH were within normal ranges, suggesting a compensatory response to reduced TRP (74–75%). Although Fanconi syndrome improved and the patient did not develop hyperglycemia, persistent glycosuria was likely attributable to empagliflozin, a sodium–glucose cotransporter 2 inhibitor. Fibroblast growth factor 23 (FGF23) also plays an important role in phosphate regulation and is clinically significant in differentiating causes of hypophosphatemic osteomalacia [16, 17]. However, FGF23 testing is not widely available and was not performed in this case. The patient’s clinical and laboratory findings, together with the dramatic improvement after switching from adefovir to entecavir, further confirmed the diagnosis of hypophosphatemic osteomalacia due to adefovir-induced Fanconi syndrome.

The mainstay of treatment is discontinuing ADV, with supplementation of phosphate, calcitriol, or calcium as needed [1, 7]. Following discontinuation or dose reduction of ADV, serum phosphate levels and clinical symptoms typically improve within 2–6 months in most cases, indicating that the key to recovery is timely withdrawal of ADV and that ADV-induced nephrotoxicity is largely reversible [2, 4, 6]. In this case, switching from adefovir to entecavir to mitigate renal and skeletal toxicity was associated with marked clinical improvement and stable chronic hepatitis B. However, if prior lamivudine resistance was present, entecavir may be suboptimal as long-term therapy because of reduced antiviral efficacy and a lower genetic barrier to resistance. Current guidelines recommend tenofovir alafenamide fumarate as a preferred option, given its potent antiviral activity and favorable renal and bone safety profile [12, 18, 19].

In summary, patients receiving long-term ADV, even at low doses, should be attentive to musculoskeletal symptoms such as bone pain or muscle weakness and undergo regular monitoring of renal function and serum ALP, phosphate, and calcium levels. Patients with preexisting renal insufficiency require more frequent monitoring. If a diagnosis of ADV-induced Fanconi syndrome with osteomalacia is established, ADV must be discontinued immediately, and phosphate supplementation initiated for symptomatic control. With early diagnosis and intervention, renal damage may be reversible [1, 6, 20].

### Learning points

Although ADV-induced Fanconi syndrome and hypophosphatemic osteomalacia are relatively rare, they severely compromise patients’ quality of life. Careful monitoring of serum phosphate and ALP, in addition to renal function, is essential for early recognition and timely management of these potentially reversible adverse drug reactions.

## Data Availability

The data supporting the finding of this study are available from the corresponding author upon reasonable request.

## Abbreviations

ADV: adefovir dipivoxil
HBV: hepatitis B virus
ALP: alkaline phosphatase
BMD: bone mineral density
MRI: magnetic resonance imaging
^99m^Tc-MDP: technetium-99m methylene diphosphonate
PTH: parathyroid hormone
25(OH)D: 25-hydroxy vitamin D
P1NP: procollagen type 1 N-terminal propeptide
CTx: C-terminal telopeptide of type 1 collagen
eGFR: estimated glomerular filtration rate
1: 25(OH)_2_D, 1,25-dihydroxyvitamin D
TRP: tubular reabsorption of phosphorus
hOAT1: human organic anion transporter 1
TDF: tenofovir disoproxil fumarate
FGF23: Fibroblast growth factor 23
